# The Moral Transition

**Published:** 1915-11

**Authors:** 


### The Moral Transition.

---

Whether or not it is as we wish, the fact remains that society today is face to face with a transition of her moral standards. And well we might ponder over what will be the moral code of tomorrow; seriously debate what will be the fundamental basis for this new code. For generations we have been under dominance of theological doctrine and our ideas of moral rectitude have been founded on this social heritage. With the greater diffusion of knowledge, there has resulted an intellectual awakening, a greater freedom of thought and independence of action. Each generation now sees the passing of some cherished mores. Prejudice and bias is rapidly receding before the challenge of scientific truth. Everywhere in the domain of natural law there have been radical changes in our conception of the meaning and interpretation of natural phenomena. And, our moral laws have proven no exception. Thoughtful men and women are asking why this and that? Why this custom, or that code? Is it in harmony with known facts? Is it right?

Well may society seek an explanation of a custom fraught with so much unhappiness and discontent, so much distress and misery, so much pauperism and crime, so much morbidity and death, as that of our present code. Well may the spirit of justice cry why this unnecessary and unnatural life?

Based on this new conception the ethics of the future will have for its purpose the development of social relations that will assure the greatest good and greatest happiness of the individual and society. Then love will know no barrier, it will neither be crushed nor bound, it will respond wholly to mutual desire and be the arbiter of its tenement. Man and woman will have a better understanding of their mutual interdependence, one on the other, and of the necessity for maintaining the highest moral relationship. New safeguards will be provided for the interest of the child and the rights of property. Unchastity and licentious living will be less prevalent. Social relations will have a greater regard for the personal liberties of others. Marriage will not be based on a religious right, it will be regarded as a social contract and entered into for mutual welfare. Society will be purged of the unfit, for overindulgence would soon exterminate the morally weak and the profligate.

The supreme test will lie in the quality of the inherited characters of the next generation. If these be ethically fit this new adjustment will mean the survival of the race; if not, then our boasted society will go down as all other civilizations have done before. The standard, therefore, will be neither science nor religion, but the ethically best, the development and survival of the moral characters.

So, again, eugenics comes to the fore as the exponent of the greatest good. This assumption of responsibility not only compels the practice of negative and positive eugenics, but also the control of the environment. Germinal cells, however good, have no significance without suitable soil for their development. This means, then, the control of education and the cultivation of moral ideals, providing of safeguards for the protection of developing youth, conservation of natural energy, knowledge of the purpose and nature of reproduction and the obligation of the individual to society. The manner and means of this direction will rest with the respective social units made manifest in the course of its transition.

---

NOT ON YOUR LIFE.

Softly the nurse smoothed the sufferer's pillow. He had been admitted only that morning, and now he looked up pleadingly at the nurse who stood at his bedside.

"An' phwat did ye say the docthor's name was, nurse dear?" he asked.

"Dr. Kilpatrick," was the reply. "He's the senior house surgeon."

The sufferer winced and pulled a wry face.

"That settles it," he muttered, firmly. "That doctor won't get a chance to operate on me."

"Why not?" asked the nurse, in surprise. "He's a very clever man."

"That's as may be," the patient said. "But me name happens to be Patrick."—*Philadelphia Public Ledger.*

